# Phytotherapy as Multi-Hit Therapy to Confront the Multiple Pathophysiology in Non-Alcoholic Fatty Liver Disease: A Systematic Review of Experimental Interventions

**DOI:** 10.3390/medicina57080822

**Published:** 2021-08-14

**Authors:** Ayokanmi Ore, Oluseyi Adeboye Akinloye

**Affiliations:** 1 Biochemistry Programme, Department of Chemical Sciences, Faculty of Natural Sciences, Ajayi Crowther University, Oyo 1066, Nigeria; 2 Department of Biochemistry, College of Biosciences, Federal University of Agriculture, Abeokuta 2240, Nigeria; oaakin@yahoo.com

**Keywords:** nonalcoholic fatty liver disease (NAFLD), metabolic dysfunction-associated fatty liver disease (MAFLD), insulin resistance, obesity, liver, steatosis, steatohepatitis, inflammation, oxidative stress, phytotherapy

## Abstract

Non-alcoholic fatty liver disease (NAFLD), or metabolic dysfunction-associated fatty liver disease (MAFLD), is a metabolic condition distinguished by fat deposition in the hepatocytes. It has a prevalence of about 25% worldwide and is associated with other conditions such as diabetes mellitus, obesity, hypertension, etc. *Background and Objectives*: There is currently no approved drug therapy for NAFLD. Current measures in the management of NAFLD include lifestyle modification such as an increase in physical activity or weight loss. Development of NAFLD involves a number of parallel hits: including genetic predisposition, insulin resistance, disordered lipid metabolism, mitochondrial dysfunction, lipotoxicity, oxidative stress, etc. Herbal therapy may have a role to play in the treatment of NAFLD, due to their numerous bioactive constituents and the multiple pharmacological actions they exhibit. Therefore, this systematic review aims to investigate the potential multi-targeting effects of plant-derived extracts in experimental models of NAFLD. *Materials and Methods*: We performed a systematic search on databases and web search engines from the earliest available date to 30 April 2021, using relevant keywords. The study included articles published in English, assessing the effects of plant-derived extracts, fractions, or polyherbal mixtures in the treatment of NAFLD in animal models. These include their effects on at least disordered lipid metabolism, insulin resistance/type 2 diabetes mellitus (T2DM), and histologically confirmed steatosis with one or more of the following: oxidative stress, inflammation, hepatocyte injury, obesity, fibrosis, and cardiometabolic risks factors. *Results*: Nine articles fulfilled our inclusion criteria and the results demonstrated the ability of phytomedicines to simultaneously exert therapeutic actions on multiple targets related to NAFLD. *Conclusions*: These findings suggest that herbal extracts have the potential for effective treatment or management of NAFLD.

## 1. Introduction

Non-alcoholic fatty liver disease (NAFLD) is a condition associated with the deposition of fat in the hepatocytes which exists in people with little or no consumption of alcohol [1]. NAFLD covers a range of conditions spanning hepatic steatosis or non-alcoholic fatty liver (NAFL) to non-alcoholic steatohepatitis (NASH), a more severe form of the disease [2]. The term non-alcoholic steatohepatitis was initially described by Ludwig et al. [3] following the discovery of a condition similar to alcoholic hepatitis in patients with no history of alcohol consumption. NAFLD is associated with multiple conditions including metabolic dysfunction, insulin resistance, steatosis, oxidative stress, inflammation, fibrosis, etc. [4].

Due to the heterogeneous nature of NAFLD, and inaccuracies in the existing terminologies, a new consensus-driven description was proposed in 2020 as ‘metabolic-dysfunction-associated fatty liver disease (MAFLD)’ [5,6]. NAFLD, or MAFLD, as it is now called, affects quite a large number of the population, and the current global prevalence was estimated to be 25% [7]. The high prevalence of this disease may be linked to excessive calorie intake relative to expenditure, a rise in sedentary lifestyle, low level of physical exercise, and consumption of unhealthy foods. Consumption of a high-fat diet (HFD) for instance, has been connected to the development of NAFLD [8]. The lipotoxicity resulting from HFD induces hepatic insulin resistance, a major player in the development of type-2 diabetes mellitus (T2DM) and NAFLD [9].

There is currently no specific treatment or approved drug therapy for NAFLD. Current measures in the management of NAFLD include lifestyle modification including an increase in physical activity, weight loss, etc. Other treatment measures are directed towards the management of the features of metabolic dysfunction, oxidative stress, inflammation, etc. involved in the pathophysiology of the disease [1,10]. While NAFLD may be managed by reasonable dietary and lifestyle modifications, regular exercise, and reduction of alcohol intake, these measures may not be effective for complete treatment or recovery. Based on available treatment options, pioglitazone is a drug of choice especially in the prevention of fibrosis in patients with diabetes [1]. Vitamin E has been useful in children and non-diabetic adults. A host of other drugs aimed at metabolic dysfunction, inflammation, and fibrosis in NASH have either failed or are still under investigation. In this systematic review, we investigate the potential multi-targeting effects of plant-derived extracts in experimental models of NAFLD.

### 1.1. The Hits in NAFLD?

NAFLD is known to progress from steatosis (deposition of fat in hepatocytes) to the development of inflammation and fibrosis, a condition called non-alcoholic steatohepatitis (NASH). In addition to steatosis, the pathological features of NASH include hepatocyte injury, inflammation, and various degrees of fibrosis. The pathogenesis of NAFLD is complex and was initially described by the two-hit hypothesis [11]. The first hit in this hypothesis being the development of steatosis, while the second hit involves a source of free radicals producing oxidative stress. In this hypothesis, lipid peroxidation is known to play a role in the development of steatohepatitis, where products of lipid peroxidation like 4-hydroxynonenal (4-HNE) and malondialdehyde (MDA), activate stellate cells in the liver, crosslinking cytokeratin to form Mallory bodies. MDA may also contribute to the inflammation seen in steatohepatitis by activating the NF-kB pathway, which regulates the expression of inflammatory cytokines.

**Figure 1:**
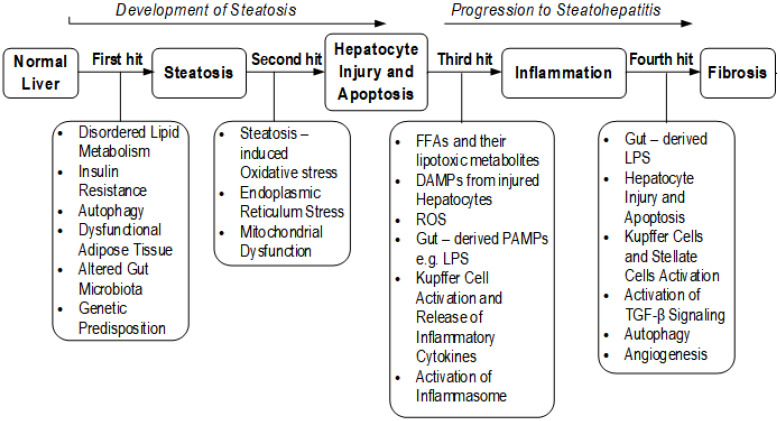
Factors contributing to the hits in NAFLD. FFA, free fatty acids; DAMP, damage-associated molecular pattern; PAMP, pathogen-associated molecular pattern; LPS, lipopolysaccharide; TGF-β, tumor growth factor-beta.

Over time, studies have identified more contributing factors indicating that NAFLD is multifactorial. The discovery of these extra contributing factors led to the multiple parallel hits hypothesis [12]. In this hypothesis, the development of NASH is due to complex interactions among a number of parallel hits: genetic predisposition, insulin resistance, disordered lipid metabolism, mitochondrial dysfunction, lipotoxicity, oxidative stress, endoplasmic reticulum (ER) stress, ethanol intake, loss of gut barrier integrity, gut-derived endotoxins like lipopolysaccharide(LPS), and altered levels of cytokines and adipokines [12,13]. Detailed mechanisms behind the development of NAFLD and the role played by each of the contributing factors have been well documented by Liu et al. [13] and Kaufmann et al. [14]. These contributing factors and their roles in NAFLD are illustrated in Figure 1.

### 1.2. Biomarkers and Criteria Used in the Diagnosis of NAFLD

NAFLD is diagnosed when patients have hepatic steatosis and any metabolic condition: overweight/obesity, diabetes mellitus, or evidence of metabolic dysregulation (MD) in lean individuals [6,10]. The definition of NAFLD in recent guidelines is characterized histologically by triglyceride accumulation within the hepatocytes exceeding 5% [15,16], in the absence of excessive alcohol consumption or any other liver disease. Various diagnostic criteria, evaluation techniques, and important biomarkers (for apoptosis, inflammation, oxidative stress, fibrosis, etc.) involved in the pathogenesis of NAFLD have been described [17,18,19,20]. Most of the biomarkers or criteria used in experimental studies are centered mainly on the factors contributing to the development and progression of the disease. Based on the new definition of the disease, Eslam et al. [6] presented criteria for diagnosis irrespective of alcohol consumption or the presence of other liver conditions. These criteria are based on evidence of hepatic steatosis (confirmed by imaging, biopsy, or blood markers) in addition to the presence of one of the types of metabolic dysregulation, such as insulin resistance/T2DM, or obesity. In addition, a criterion for diagnosis of MAFLD in lean or non-obese patients was proposed to include the presence of steatosis with a minimum of two metabolic risk abnormalities listed in Table 1.

### 1.3. Phytotherapy

Phytotherapy or phytomedicine or herbal medicine refers to the application of extracts of natural origin as medicines or health-promoting agents [21]. It involves the use of herbs as medicine in the treatment or prevention of diseases both in humans and animals [22]. In folk practices, herbal medicines are composed of extracts from one or multiple plant parts, thus phytoconstituents are highly variable with numerous biologically active constituents. The therapeutic effects of herbs or phytomedicines are directed against both the causes and symptoms of a disease. In recent years, research efforts have been directed toward the exploitation of the multi-targeting potentials embedded in herbal medicines in the treatment of diseases with multi-factorial causes [23,24]. These multi-target phytotherapeutic effects are currently being investigated in the treatment of numerous diseases including malaria [25], Alzheimer’s disease (AD) [26], heart disease [27], insulin resistance [28], T2DM [29], and NAFLD [30].

### 1.4. Rationale and Objectives

The multi-factorial or heterogeneous nature of NAFLD suggests that it may neither be managed as a single condition nor effectively treated with a single therapeutic agent. Therefore, phytomedicines or herbal medicines may have important roles to play due to their numerous bioactive constituents and multiple therapeutic principles they display in experimental studies. The objective of this study is to provide a systematic review of available data on the efficacy and multiple therapeutic mechanisms exhibited by phytomedicines in experimental models of NAFLD.

## 2. Methods

This systematic review was carried out according to the Preferred Reporting Items for Systematic Review and Meta-Analyses (PRISMA) guidelines for systematic reviews and meta-analysis [31] (see Table S1 Checklist; Figure S1).

### 2.1. Eligibility Criteria

Inclusion criteria:

(a) Articles that were published in English,
(b) Animal studies,
(c) NAFLD model induced by diet
(d) Studies assessing the effectiveness of plant-derived extracts or fractions or polyherbal mixtures in the treatment of NAFLD
(e) Studies evaluating at least disordered lipid metabolism, insulin resistance/T2DM, and histologically confirmed steatosis
(f) Studies with one or more of the following: oxidative stress, inflammation, hepatocyte injury, obesity, fibrosis, and cardiometabolic risks (in addition to the pathological conditions in (e)).

Exclusion criteria:

(a) Studies conducted in vitro,
(b) Human studies
(c) Reviews,
(d) NAFLD not induced by diet
(e) Studies involving the use of isolated or single phyto-compound to treat NAFLD
(f) Studies not meeting the inclusion criteria stated above.

### 2.2. Information Sources

Data sources include published findings searched via databases and web search engines like PubMed Central (PMC), Scopus, DOAJ, BASE, PubMed, ScienceDirect, and Google Scholar. These databases were searched for findings published until April 2021.

### 2.3. Search Strategy

The strategies include the use of a combination of the following terms: “plant extracts AND non-alcoholic fatty liver disease”; “plant extracts AND non-alcoholic steatohepatitis”; “leaf extracts AND non-alcoholic fatty liver disease”; “leaf extracts AND non-alcoholic steatohepatitis”; “seed extracts AND non-alcoholic fatty liver disease”; “seed extracts AND non-alcoholic steatohepatitis”; “stem extracts AND non-alcoholic fatty liver disease”; “stem extracts AND non-alcoholic steatohepatitis”; “root extracts AND non-alcoholic fatty liver disease”; “root extracts AND non-alcoholic steatohepatitis”; “plant extracts AND NAFLD”; “plant extracts AND NASH”; “leaf extracts AND NAFLD”; “leaf extracts AND NASH”; “seed extracts AND NAFLD”; “seed extracts AND NASH”; “stem extracts AND NAFLD”; “stem extracts AND NASH”; “root extracts AND NAFLD”; “root extracts AND NASH”. References from the retrieved articles were further searched to obtain more studies.

### 2.4. Study Selection

The full-text article selection criteria included:

(a) Animal models,
(b) Assessments of the effectiveness of plant extracts in NAFLD model
(c) Full-text articles in English
(d) Evaluation of disordered lipid metabolism, steatosis, insulin resistance (HOMA-IR), with one or more of the following: oxidative stress, inflammation, hepatocyte injury, obesity, fibrosis, and cardiometabolic risks.

### 2.5. Data Collection

Authors independently extracted from each of the included studies information on the authors, animal model, intervention (dose of extract and duration of the study), outcome measures regarding markers/indices (insulin resistance (HOMA-IR), disordered lipid metabolism, steatosis, with or without oxidative stress, inflammation, hepatocyte injury, obesity, fibrosis, and cardio-metabolic data) for both extract-treated and NAFLD controls. In studies with multiple interventions, only data from the extract-treated and NAFLD control groups were considered in the systematic review.

## 3. Results

### 3.1. Study Selection

**Figure 2:**
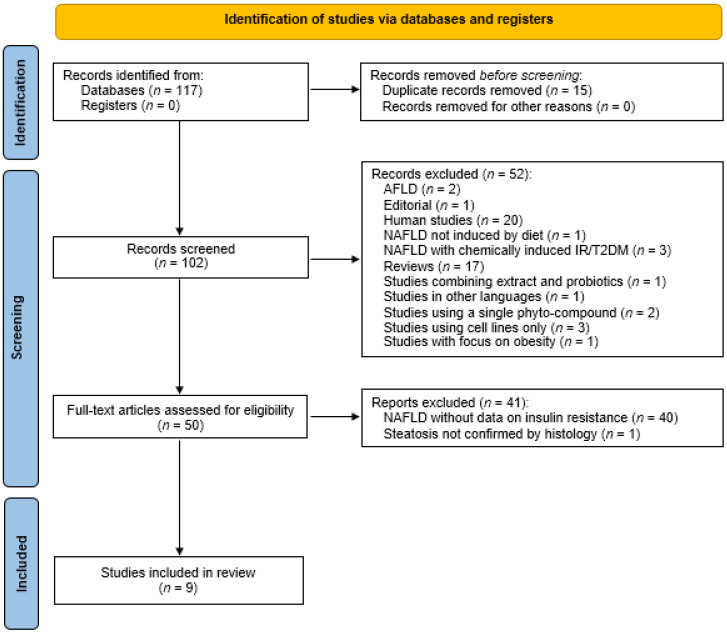
Flowchart depicting the screening and selection of studies included in the systematic review in accordance with the preferred reporting items for systematic reviews and meta-analyses (PRISMA) statement [31]. NAFLD, non-alcoholic fatty liver disease; AFLD, alcoholic fatty liver disease; IR, insulin resistance; T2DM, type-2 diabetes mellitus.

As of April 2021, our search identified 117 records across the databases searched. After careful evaluation of the article type, titles, and abstracts, we eliminated 15 duplicate records. The 102 records remaining were screened, resulting in the exclusion of 52 records based on the reasons listed in Figure 2. Thereafter, 50 articles were assessed for eligibility; 40 out of these were excluded for not containing data on insulin resistance/type2 diabetes mellitus and 1 article without histologically confirmed steatosis. Out of the 50 assessed for eligibility, 9 articles matching our inclusion criteria were included in the systematic review (see Figure 2).

### 3.2. Study Characteristics

Of the 9 included studies, all reported the effect of phytomedicine tested in each case on lipometabolism, glycometabolism (or insulin resistance), and hepatic steatosis. Out of these, 6 reported oxidative stress, 7 reported hepatocyte injury, 4 reported inflammation, 8 reported body weight gain/obesity, 3 reported fibrosis, and 2 contained data on atherosclerosis/cardiometabolic risk factors. The characteristics of the studies included in the systematic review and biomarkers measured across the included studies are presented in Table 2.

## 4. Discussion

**Figure 3:**
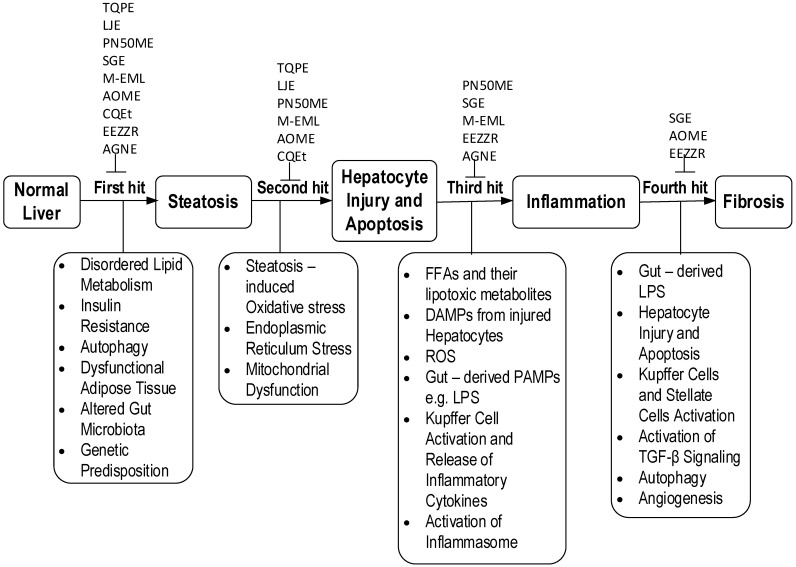
Mechanisms of action of extracts/fractions on NAFLD. TQPE, *Trapa quadrispinosa* pericarp extract; LJE, *Leonurus japonicus* ethanol extract; PN50ME, *Phyllanthus niruri* 50% methanol in water extract; SGE, *Salvia miltiorrhiza* root and fruit of *Gardenia jasminoides* extracts; M-EML, Methanol extract from *Erica multiflora* leaf; AOME, *Alisma orientalis* methanolic extract; CQEt, *Cissus quadrangularis* stem extract (CQEt); EEZZR, Ethanol extract of *Zingiber zerumbet* rhizome; AGNE, *Angelica gigas* Nakai extract (AGNE).

The outcome of this systematic review reveals the multi-targeting effects of nine plant-derived extracts on the various pathophysiological components of NAFLD as summarized in Table 2. These include alleviation of dyslipidemia, insulin resistance, steatosis, oxidative stress, hepatic injury, inflammation, and fibrosis among other effects as discussed in the following subsections. The potential mechanisms of protection exhibited by these extracts/fractions against NAFLD are summarized in Figure 3.

### 4.1. Trapa quadrispinosa Pericarp Extract (TQPE) Exerts Multiple Therapeutic Hits on NAFLD

*Trapa quadrispinosa* (or water caltrop) is a plant that has been applied for years in Chinese folk medicine in the treatment of diabetes mellitus. TQPE is rich in polyphenols and at 30 mg/kg, it exhibited multiple effects against HFD induced NAFLD in mice [32]. TQPE effectively alleviated dyslipidemia in this study. Dyslipidemia in NAFLD patients is characterized by high triglycerides and high LDL-c levels and low HDL-c levels [41]. TQPE significantly decreases plasma total cholesterol, triglycerides, and LDL-cholesterol coupled with significant improvement in HDL-c level. LDL is one of the five major groups of lipoprotein involved in the transport of lipids around the body. LDL delivers fat molecules (mainly cholesterol, phospholipids, and triglycerides) to cells and a high level of circulating LDL-c is a well-established risk factor for atherosclerosis [41], a comorbidity in NAFLD. On the other hand, high HDL level as observed in TQPE treatment correlates with reduced risk of atherosclerosis.

TQPE intervention also causes a significant increase in hepatic expression of p-AMPK and p-ACC with a decline in SREBP expression. 5′AMP-activated protein kinase (AMPK) is an enzyme that plays a role in metabolic regulation. It is involved in the activation of fatty acid and glucose uptake and their oxidation when cellular energy is depleted. Activated AMPK is known to promote energy production, and suppresses energy-requiring processes [42]. SREBPs are transcription factors that regulate the expression of genes involved in lipid synthesis while Acetyl-CoA carboxylase (ACC) catalyzes the rate-limiting step in fatty acid biosynthesis, involving the carboxylation of acetyl-CoA in the formation of malonyl-CoA [43,44]. Activation of SREBP-1 due to high blood insulin level plays a key role in the induction of lipogenesis that leads to hepatic steatosis. A high level of circulating insulin activates mTORC1, leading to the increased production of SBREP-1c which facilitates the storage of fatty acids as triglycerides [45]. Activation of AMPK on the other hand leads to inhibition of fatty acid synthesis via a reduction in the transcriptional activation of SREBP. TQPE also restored insulin balance and insulin sensitivity in mice via a decrease in p-IRS1/IRS1 and p-Akt/Akt ratio. Impaired IRS-1 is associated with NAFLD and T2DM. Protein kinase B (also known as Akt) plays a vital role in insulin signal transduction [46]. Activated Akt decreases lipogenesis and increases hepatic glucose uptake and glycogenesis. In this study, TQPE increases hepatic Akt activation thereby enhancing insulin sensitivity in mice. TQPE also alleviates elevated body weight, hepatic steatosis, oxidative stress, and hepatocellular damage in HFD-fed mice.

### 4.2. Leonurus japonicus Ethanol Extract (LJE) and Its Multiple Hit Effects on NAFLD

*Leonurus japonicus* Houtt. (Lamiaceae), or motherwort, is a plant with important medicinal values and is commonly found around Asia [47]. The aerial part of *L. japonicus* has exhibited various therapeutic effects such as anticancer and neuroprotection [33,48]. In a study carried out by Lee et al. [33], *Leonurus japonicus* ethanol extract (LJE) supplementation at 100 and 200 mg/kg for 12 weeks exhibited multiple therapeutic effects on NAFLD in mice. These effects include suppression of hepatic lipid accumulation, insulin resistance, weight gain, oxidative stress, and alleviation of hepatocyte injury. LJE positively influenced lipid metabolism in HFD induced NAFLD via a decrease in circulating levels of cholesterol, triglycerides, LDL-cholesterol, and a decrease in hepatic triglycerides level. LJE also significantly improved hepatic expression of p-AMPK and peroxisome proliferator-activated receptor-alpha (PPAR-α). PPARα, highly expressed in the liver plays a vital role in the regulation of ketogenesis, fatty acid uptake, beta oxidation, triglyceride turnover, and regulation of inflammation [49]. The hepatic expression of PPARα is known to decrease in humans with NAFLD and increase in response to various interventions [50]. Also, histological assessment of hepatocytes shows a reduction in steatosis in response to LJE intervention. LJE also causes a significant decrease in circulating levels of glucose and insulin with the alleviation of insulin resistance. LJE alleviates oxidative and hepatocellular damage in mice as indicated by a decrease in hepatic MDA content and serum activities of ALT, AST, and LDH.

### 4.3. Multiple Effects of Phyllanthus niruri 50% Methanol in Water Extract (PN50ME) on NAFLD

*P. niruri* is a herb applied in traditional medicine to treat or manage a number of pathological conditions like hepatitis, tuberculosis, diabetes, bronchitis, asthma, tumors, etc. [51]. It contains polyphenols that have been linked with antioxidant and hepatoprotective activities of the plant [34,52]. Al Zarzour et al. [33] demonstrated the multiple therapeutic effects of 1000 mg/kg *Phyllanthus niruri* 50% methanol in water extract (PN50ME) on NAFLD. PN50ME significantly suppressed dyslipidemia, steatosis and hepatocyte ballooning, insulin resistance, oxidative stress, hepatocyte injury, inflammation, and obesity in a rat model of HFD-induced NAFLD. In addition, PN50ME also reduces atherosclerosis risk in rats as indicated by significantly lower Castelli’s Risk Index-1 (CRI-I); Castelli’s Risk Index-2, and atherogenic coefficient.

### 4.4. Multiple Therapeutic Actions of Combinations of Salvia miltiorrhiza Root and Fruit of Gardenia jasminoides Extracts (SGE)

The combination of *Salvia miltiorrhiza* root and fruit of *Gardenia jasminoides* extracts or SGE exerts multiple therapeutic effects on NAFLD in rat models as reported by Tan et al. [35]. The dried root of *Salvia miltiorrhiza* is a component of prescriptions for chronic liver disease, hyperlipidemia, and heart disease in traditional Chinese medicine (TCM) [35,53]. The fruit of *Gardenia jasminoides* on the other hand is a prescription for detoxification. An important property of TCM is the combination of several herbs to treat many abnormalities associated with chronic diseases [54]. Tan et al. [35], demonstrated the multiple therapeutic effects of SGE against hyperlipidemia, hepatic steatosis, insulin resistance, inflammation, hepatocellular injury, obesity, and fibrosis-related to NAFLD. SGE regulated lipid metabolism in this model via a decrease in serum FFA, TG, TC, and LDL-c; increase serum HDL-c and adipose tissue expression of leptin. The previous report indicated that leptin administration may alleviate insulin resistance in patients with lipodystrophy [55]. Leptin is known to play a key role in the regulation of energy homeostasis mainly via suppressing appetite [56]. It is expressed mainly in adipose tissue and secreted into the circulation [57]. SGE also regulated glycometabolism in this NAFLD model by relieving insulin resistance, thereby causing a decrease in serum concentrations of insulin and glucose.

SGE further suppressed the expression of adipose tissue expression of the adipokines, TNF-α and IL-6. Adipocytokines are derived majorly from the adipose tissue and they play major roles in the initiation of inflammation and other metabolic processes in the pathogenesis of NAFLD [58,59].

### 4.5. Multiple Therapeutic Effects of Methanol Extract from Erica multiflora Leaf (M-EML) on NAFLD

*Erica multiflora* is a medicinal flowering plant that belongs to the family *Ericaceae*. It exhibits a wide range of bioactivities, including anti-inflammatory, hypolipidemic, antioxidant, etc. [60,61]. Khlifi et al. [36] show that M-EML is rich in polyphenols particularly kaempferol-3-O-glucoside and quercetin-3-O-glucoside. They further demonstrated the multiple therapeutic effects of M-EML on NAFLD in rats. M-EML alleviated hyperlipidemia, steatosis, insulin resistance, oxidative stress, hepatocyte injury, inflammation, obesity, and cardiometabolic risk related to NAFLD in rats. M-EML positively influenced lipid metabolism as indicated by a significant decrease in plasma lipase activity and levels of TG, TC, LDL-c, and VLDL-c, coupled with an increase in HDL-c level. M-EML also alleviates high plasma glucose and insulin and insulin resistance and hepatic steatosis (area of liver steatosis). M-EML alleviated oxidative stress induced by HFD as indicated by a significant decrease in hepatic MDA content coupled with an increase in activities of SOD, CAT, and GPx. Blood activities of biomarkers of hepatic function ALT, AST, and ALP, and total and direct bilirubin levels were significantly lower in M-EML treated rats, indicating prevention of hepatocellular damage. M-EML also alleviates inflammation, excessive weight gain, and cardiometabolic risks associated with NAFLD in the rat.

### 4.6. Alisma orientalis Methanolic Extract (AOME) Alleviate Hyperlipidemia, Hepatic Steatosis, Insulin Resistance, Oxidative Stress, Hepatocyte Injury, and Obesity Associated with NAFLD

*Alisma orientale,* also known as Asian water plantain, is a flowering plant that belongs to the genus Alisma native to Asia. The rhizomes of *A. orientale* is a very important TCM and kampo Japanese medicine used in the treatment of numerous conditions including inflammation, hypertension, kidney failure, hyperlipidemia, hyperglycemia, etc. [62]. The major constituents of *A. orientale* are terpenoids and small quantities of flavonoids, and alkaloids [63].

Hong et al. [37] reported the ability of *Alisma orientalis* methanolic extract (AOME) to alleviate NAFLD caused by a high-fat diet in rats. AOME significantly lowers serum and hepatic total cholesterol and triglycerides levels coupled with a significant reduction in weight gain and hepatic steatosis as shown in H&E-stained sections. In addition to its effects on lipometabolism, AOME also exerts a positive influence on glycometabolism as indicated by a significant decrease in fasting serum glucose and insulin levels, decrease in insulin resistance index coupled with enhanced insulin sensitivity. AOME also alleviates oxidative stress related to NAFLD in rats as indicated by a decrease in hepatic MDA level and increase in activity or SOD. It also prevented hepatocellular damage as implied by a significant decrease in serum activities of ALT and AST. Masson’s trichrome staining of liver sections also revealed that AOME significantly prevented fibrogenesis in HFD fed rats.

### 4.7. Cissus quadrangularis Stem Extract (CQEt)

*Cissus quadrangularisis* is a perennial plant with numerous medicinal properties that are well distributed in the tropics. Several pharmacological properties of *C. quadrangularisis* have been reported, including anti-obesity, anti-diabetic, antioxidant, analgesic, anti-inflammatory, etc. [64,65]. Chidambaram et al. [38] supplemented HFFD with CQEt at a 10 g/100 g diet to alleviate NALFD in the albino rat model. In this study, CQEt alleviated hyperlipidemia, hepatic steatosis, insulin resistance, oxidative stress, hepatocyte injury, and obesity-associated with NAFLD. CQEt alleviates hyperlipidemia as indicated by a significant decrease in the hepatic level of FFAs, TC, and TG. It also prevented the micro and macrovesicular fatty changes seen in HFFD fed rats as indicated in H&E and ORO stained liver sections. Insulin resistance was significantly relieved by CQEt supplementation as indicated by a decrease in plasma insulin and glucose and insulin resistance indices (HOMA-IR, QUICKI, FIRI). CQEt also protected against oxidative stress as shown by a significant decrease in levels of hepatic oxidative stress markers (TBARS, lipid hydroperoxides, protein carbonyls) and a significant increase in activities of enzymic antioxidants (SOD, CAT, GPx,). Hepatocellular integrity was preserved by CQEt supplementation as indicated by a decrease in plasma activities of liver function biomarkers (ALT, AST, GGT, and ALP).

### 4.8. Ethanol Extract of Zingiber zerumbet Rhizome (EEZZR) Attenuates NAFLD by Multiple Mechanism

*Zingiber zerumbet* (wild ginger or bitter ginger) is an aromatic and tuberose plant, which belongs to the ginger family. In folk medicine, it is applied in the treatment of inflammation, indigestion, diarrhea, fever, and pain [66]. It has exhibited a number of activities, including antioxidant, anti-cancer, anti-inflammatory, and used to alleviate osteoarthritis [67,68]. Chang et al. [39] show that *Ethanol extract of Zingiber zerumbet* rhizome (EEZZR) alleviates dyslipidemia and hepatic steatosis in HFD fed hamsters via a decrease in plasma total cholesterol and triglycerides, plasma LDL-c, plasma FFAs, and plasma HDL-c. EEZZR also caused a significant decrease in hepatic expression of SREBP-1c, acetyl CoA carboxylase 1 (ACC1), fatty acid synthase (FAS), and stearoyl CoA desaturase 1 (SCD-1) (see Table 2). SREBP-1c is a major regulator of fatty acids synthesis and plays a significant role in the NAFLD where its expression is often very high [69]. FAS catalyzes fatty acid synthesis, mainly palmitic acid, whereas, ACC1 is a cytoplasmic biotin-dependent multienzyme system that catalyzes the irreversible carboxylation of acetyl-CoA to malonyl-CoA in the biosynthesis of fatty acid. High expression of ACC1 is associated with NAFLD and was proposed as a potential treatment target for the disease where its inhibition reverses insulin resistance and NAFLD [70,71].

SCD-1 is an enzyme-based in the endoplasmic reticulum that catalyzes the rate-limiting step in the production of unsaturated fatty acids from stearoyl-CoA and palmitoyl-CoA. Elevated activity or expressions of FAS and SCD-1 has been reported in NAFLD [72,73]. However, intervention with EEZZR in this study significantly suppressed the high expressions of SREBP-1c, ACC1, FAS, and SCD-1 associated with NAFLD in hamsters. Furthermore, EEZZR also alleviates the NAFLD-associated decrease in expression of PPARα, CPT-1, ACO, and ACOX1. Carnitine palmitoyltransferase I (CPT-1) is a mitochondrial enzyme that catalyzes the formation of acylcarnitines via the transfer of the acyl group of a long-chain fatty acyl-CoA to L-carnitine. Whereas, acyl-CoA oxidase (ACO) is an enzyme involved in the hepatic peroxisomal β-oxidation system and acetyl-CoA produced from this system has been implicated in the promotion of steatosis in mice [74].

### 4.9. Angelica gigas Nakai Extract (AGNE) Alleviate Dyslipidemia, Hepatic Steatosis, Insulin Resistance and Inflammation Related to NAFLD

*Angelica gigas Nakai*, also known as ‘Korean danggui’ has been applied in folk medicine to treat numerous diseases such as anemia, pain, gynecological diseases, diabetes, and used as a sedative [75]. Other reported bioactivities include anti-cancer, neuroprotective, anti-inflammatory, anti-osteoporosis, etc. [76,77,78,79]. *A. gigas Nakai* is rich in coumarins mainly nodakenin, decursin, and decursinol angelate [80]. Bae et al. [40] showed the multi-therapeutic effects of *Angelica gigas Nakai* extract (AGNE) against NAFLD in mice fed with a high-fat diet. AGNE positively modulates biomarkers related to lipid metabolism. There was a significant decrease in serum leptin, hepatic TG, serum TG, TC, and LDL levels in response to AGNE intervention. There was also a significant decrease in hepatic expression of FAS, SREBP1, CD36, and SCD-1, while the expressions of Sirt1, p-ACC, and p-AMPK increased significantly in response to AGNE intervention (see Table 2).

Cluster of differentiation 36 (or CD36), or fatty acid translocase (FAT), is a transmembrane scavenger receptor in tissues involved in the uptake of long-chain fatty acids (FA) and contributes to the accumulation of lipids and metabolic dysfunction in conditions associated with high fat supply [81], such as in HFD induced-obesity and NAFLD [82,83]. AGNE intervention significantly disrupts the expression of CD36 in the liver of mice which agrees with a report on alleviation of fatty liver and improvement in insulin sensitivity following hepatocyte-specific disruption of CD36 function in HFD-fed mice [84]. Stearoyl-CoA desaturase-1 (SCD-1) is a Δ-9 desaturase that converts saturated fatty acids into monounsaturated fatty acids. SCD-1 is important in metabolism and metabolic diseases [85], including obesity and NAFLD [86], where its activity has been found to increase. As shown by Bae et al. [40], AGNE significantly depresses the expression of this enzyme in HFD-fed mice. NAD-dependent deacetylase sirtuin-1, or sirtuin-1 (Sirt1), is currently one of the targets of interest in the treatment of NAFLD [87] and related conditions. Sirt1 is known to play beneficial roles in NAFLD via regulation of hepatic lipid metabolism, inflammation, and oxidative stress by deacetylation of relevant transcriptional regulators [88]. Upregulation of Sirt1 as seen in AGNE intervention has been reported to alleviate NAFLD in mice [89].

## 5. Conclusions

The present systematic review suggests that plant-based extract may have significant positive effects on non-alcoholic fatty liver disease. These therapeutic effects are made possible by simultaneously exerting their actions on multiple targets. Herbal interventions covered in this review were able to alleviate the major contributing factors in the development of NAFLD. The outcome of this study indicated that the nine extracts are capable of countering the events related to the first hit (the major hit) leading to the development of steatosis. Other hits may also be simultaneously targeted by some of these extracts according to this study. However, further studies are required on phytoconstituents, their quantities, as well as their safety and efficacy in the treatment of NAFLD in large-scale clinical trials.

## Figures and Tables

**Table 1:** Cardiometabolic and MAFLD risk abnormalities. Adapted from Eslam et al. [6].

Metabolic Risk Abnormalities	Values
Waist circumference	≥102/88 cm in Caucasian men and women (or ≥90/80 cm in Asian men and women)
Blood pressure	≥130/85 mmHg or specific drug treatment
Plasma triglycerides	≥150 mg/dL (≥1.70 mmol/L) or specific drug treatment
Plasma HDL-cholesterol	<40 mg/dL (<1.0 mmol/L) for men and <50 mg/dL (<1.3 mmol/L) for women or specific drug treatment
(a) Fasting glucose levels or (b) 2-h post-load glucose levels or (c) HbA1c	100 to 125 mg/dL (5.6 to 6.9 mmol/L) 140 to 199 mg/dL (7.8 to 11.0 mmol) 5.7% to 6.4% (39 to 47 mmol/mol)
HOMA-IR score	≥2.5
Plasma hs-CRP level	>2 mg/L

HbA1c, glycated hemoglobin; HOMA-IR, Homeostasis model assessment of insulin resistance; hs-CRP, high-sensitivity C-reactive protein.

**Table 2:** Characteristics of the included studies.

Phytomedicine	Dosage	NAFLD Model	Study Duration	Effect on Lipometabolism	Effect on Glycometabolism/ Insulin Resistance	Effect on Hepatic Steatosis	Effect on Oxidative Stress	Effect on Hepatocyte Injury	Effect on Inflammation	Effect on Obesity	Effect on Fibrosis	Effect on Cardiometabolic Risk	Reference
*Trapa quadrispinosa* pericarp extract (TQPE)	30 mg/kg/d TQPE (p.o.)	HFD/ICR mice	12 weeks (therapy from week 5)	↓P-TG ↓P-TC ↓P-LDL-c ↑P-HDL-c ↑p-AMPK/AMPK ↓SREBP/β-Actin ↑p-ACC/ACC	↓S-INS ↓HOMA-IR ↑p-IRS1/IRS1 ↑p-Akt/Akt	↓Steatosis (HE)	↓MDA ↑SOD	↓P-ALT ↓P-AST	n/a	↓BW gain	n/a	n/a	[32]
*Leonurus japonicus* ethanol extract (LJE)	100 or 200 mg/kg/d LJE p.o.	HFD/male C57BL/6 mice	14 weeks	↓S-TG; ↓L-TG ↓S-TC; ↓L-TC ↓S-LDL-c ↓SREBP (ns) ↑p-AMPK/AMPK ↑PPAR-α	↓S-INS ↓S-GLU ↓HOMA-IR	↓Steatosis (HE)	↓MDA	↓S-ALT ↓S-AST ↓S-LDH	n/a	↓BW gain	n/a	n/a	[33]
*Phyllanthus niruri* 50% methanol in water extract (PN50ME)	1000 mg/kg bw/d PN50ME p.o.	HFD/male Sprague–Dawley rats	8 weeks (therapy from week 5)	↓S-FFA ↓S-TC; ↓L-TC ↓L-TG ↓S-LDL	↓S-INS ↓S-GLU ↓HOMA-IR	↓Steatosis (HE) ↓Hepatocyte Ballooning	↓MDA	↓S-ALT ↓AST/ALT	↓inflammation score (HE)	↓BW gain	n/a	↓CRI-I ↓CRI-II ↓AC	[34]
*Salvia miltiorrhiza root* and fruit of *Gardenia* *jasminoides* extracts (SGE)	2 g/kg bw/d SGE p.o.	HFD/Male Sprague-Dawley	10 weeks; therapy from week 7–10	↓S-FFA ↓S-TG; ↓L-TG ↓S-TC ↓S-LDL-c ↑S-HDL-c ↑AT-Leptin	↓S-INS ↓S-GLU ↓HOMA-IR	↓Steatosis (HE & ORO)	n/a	↓S-ALT ↓S-AST	↓AT-TNF-α ↓AT-IL-6	↓BW gain ↓Visceral fat mass	↓Fibrosis (M3T)	n/a	[35]
Methanol extract from *Erica multiflora* leaf (M-EML)	250 mg/kg bw/d p.o.	HFHFD/male Wistar rats	8 weeks (therapy from week 5)	↓P-TG ↓P-TC ↓P-LDL-c ↓P-VLDL-c ↓P-Lipase ↑P-HDL-c	↓P-INS ↓P-GLU ↓HOMA-IR	↓Steatosis (HE & ORO)	↓MDA ↑SOD ↑CAT ↑GPx	↓B-ALT ↓B-AST ↓B-ALP ↓P-TB ↓P-DB	↓NO ↓Lysosomal activity ↓P-TNF-α ↓P-IL-6	↓BW gain	n/a	↓CRI ↓AIP ↓AI	[36]
*Alisma orientalis* methanolic extract (AOME)	150,300 and 600 mg kg^−1^)	HFD/Male Sprague-Dawley rats	12 weeks, therapy from week 7	↓S-TG; ↓L-TG ↓S-TC; ↓L-TC	↓FSG ↓FSI ↑ISI ↓IRI	↓Steatosis (HE)	↓S-MDA ↑S-SOD	↓S-ALT ↓S-AST	n/a	BW gain (ns)	↓Fibrosis (M3T)	n/a	[37]
*Cissus quadrangularis* stem extract (CQEt)	CQEt (10 g/100 g diet	HFFD/male Wistar albino rats	60 days, therapy from day 16	↓L-FFAs ↑L-PL ↓L-TG ↓L-TC	↓P-INS ↓P-GLU ↓HOMA-IR ↓QUICKI ↓FIRI	↓Steatosis (HE & ORO	↓L-TBARS ↓L-LHP ↓L-Protein carbonyls ↑SOD ↑CAT ↑GPx	↓P-ALT ↓P-AST ↓P-GGT ↓P-ALP	n/a	↓BW gain	n/a	n/a	[38]
Ethanol extract of *Zingiber zerumbet* rhizome (EEZZR)	200, and 300 mg/kg	HFD/Male Golden Syrian hamsters	10 weeks, therapy from week 3	↓L-TC; ↓P-TC ↓P-TG; ↓L-TG ↓P-LDL-c ↓P-FFAs ↑P-HDL-c ↓L-SREBP-1c ↓L-ACC1 ↓L-FAS ↓L-SCD-1 ↑L-PPARα ↑L-CPT-1 ↑L-ACO ↑L-ACOX1	↓P-INS ↓P-GLU ↓HOMA-IR	↓Steatosis (HE	n/a	n/a	↓L-TNF-α ↓L-IL-6 ↓L-MCP1 ↓F4/80	↓BW gain	↓A-SMA	n/a	[39]
*Angelica gigas* Nakai extract (AGNE)	40 mg/kg)	HFD/c57BL6/J mice	16 weeks.	↓S-Leptin ↓L-TG ↓S-TG ↓S-TC ↓S-LDL S-HDL (ns) ↓L-FAS/β-actin ↓SREBP1/LaminB ↓L-CD36/β-actin ↓L-SCD-1/β-actin ↑Sirt1/β-actin ↑p-AMPK/AMPK ↑L-p-ACC/ACC	↓S-INS ↓S-GLU ↓HOMA-IR ↓B-GLU (GTT) ↓B-GLU (ITT) ↑S-Adiponectin ↑p-Akt/Akt	↓Steatosis (HE, ORO)	n/a	n/a	↓S-TNF-α ↓S-IL-6 ↓S-MCP1 ↓L-F4/80	n/a	n/a	n/a	[40]

AC, Atherogenic Coefficient ((TC-HDL)/HDL); ACC, acetyl CoA carboxylase; ACO, acyl-CoA oxidase, atherogenic index; AIP, atherogenic index of plasma; Akt, PKB (Protein kinase B); ALP, alkaline phosphatase; ALT, alanine aminotransferase; ALT, alanine aminotransferase; AMPK, AMP-activated protein kinase; AST, aspartate aminotransferase; AT-, adipose tissue; B-, blood; bw, body weight; CD, cluster of differentiation; CRI, coronary risk index; CRI-I, Castelli’s Risk Index-1 (TC/HDL); CRI-II, Castelli’s Risk Index-2 (LDL/HDL); DB, direct bilirubin; FIRI, fasting insulin resistance indices; FSG, fasting serum glucose; FSI, fasting serum insulin; GLU, glucose; GTT, Glucose tolerance test; HDL, high density lipoprotein; HE, hematoxylin and eosin; HFD, high fat diet; HFHFD, high fat, high fructose diet; HOMA-IR, homeostatic model assessment of insulin resistance; IL, interleukin; INS, insulin; IRI, insulin resistance index; ISI, insulin sensitivity index; ITT, insulin tolerance test; L-, liver; LDL-c, low density lipoprotein cholesterol; LHP, lipid hydroperoxides; M3T, masson’s trichrome stain; MCP1, monocyte chemoattractant protein 1; MDA, malondialdehyde; ns, not significant (or no significant change); ORO, oil red O; p-, phosphorylated; P-, plasma; p.o., per os; PL, phospholipids; PPAR-α, peroxisome proliferator-activated receptor-alpha; QUICKI, quantitative insulin sensitivity check index; S-, serum; SCD, stearoyl CoA desaturase; SOD, superoxide dismutase; SREBP, sterol regulatory element binding protein; SREBP-1c, sterol regulatory element-binding proteins-1c; TB, total bilirubin; TBARS, thiobarbituric acid reactive substances; TC, total cholesterol; TG, liver triglycerides; VLDL, very-low-density lipoprotein; wt, weight.

## Data Availability

Not applicable.

## Supplementary Materials

The following are available online at https://www.mdpi.com/article/10.3390/medicina57080822/s1, Table S1: PRISMA 2020 Checklist. Figure S1: PRISMA 2020 flow diagram for new systematic reviews which included searches of databases and registers only.
