# Supplementary material for: Phytotherapy as Multi-Hit Therapy to Confront the Multiple Pathophysiology in Non-Alcoholic Fatty Liver Disease: A Systematic Review of Experimental Interventions
Source: Medicina (Kaunas). 2021 Aug 14;57(8):822. doi: 10.3390/medicina57080822 (PMC8400978; doi:10.3390/medicina57080822)
Supplement: Supplementary file 1 [file medicina-57-00822-s001.zip › Figure S1.pdf]

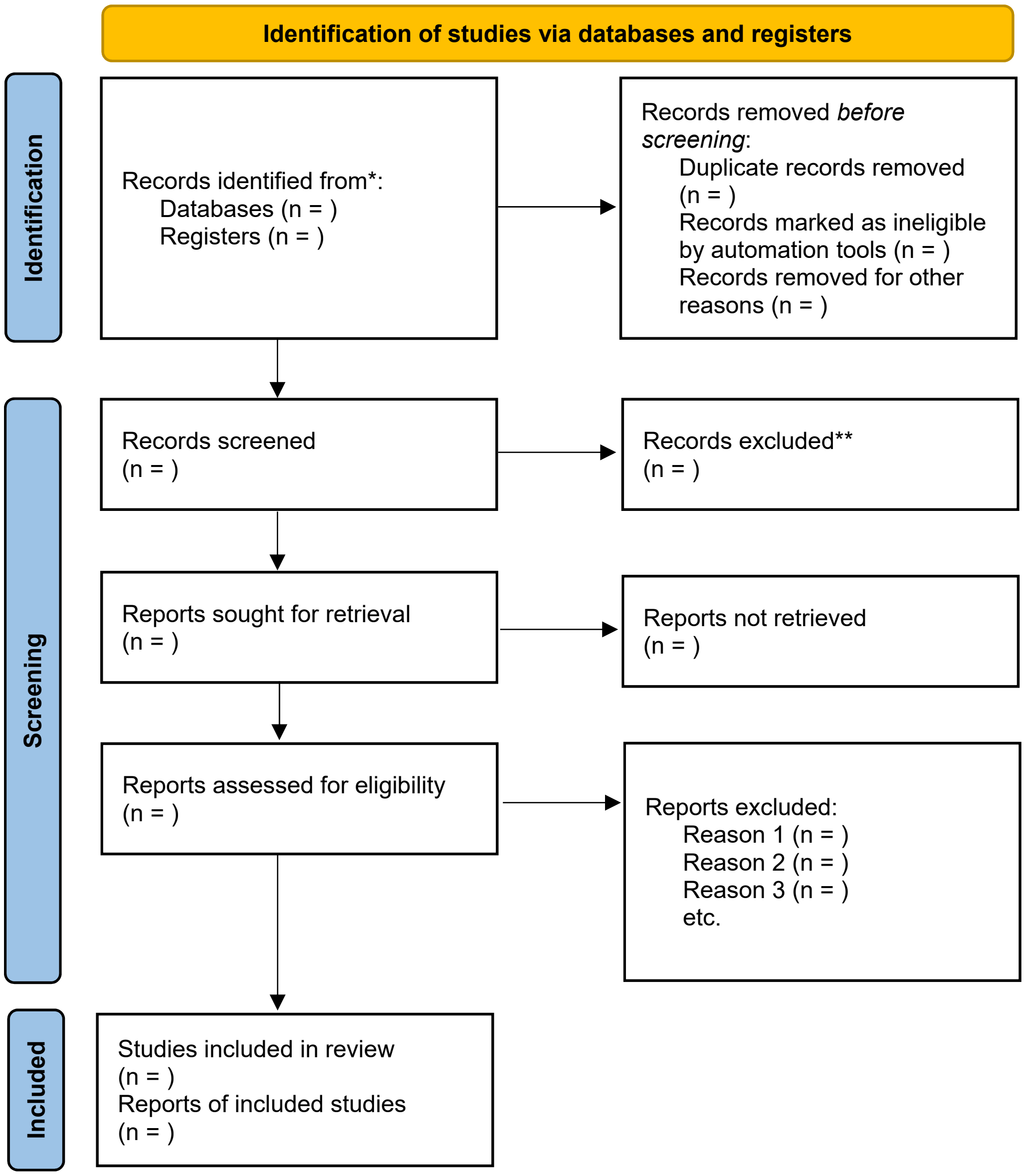

Figure S1: PRISMA 2020 flow diagram for new systematic reviews which included searches of databases and registers only

*Consider, if feasible to do so, reporting the number of records identified from each database or register searched (rather than the total number across all databases/registers).

**If automation tools were used, indicate how many records were excluded by a human and how many were excluded by automation tools.

From: Page MJ, McKenzie JE, Bossuyt PM, Boutron I, Hoffmann TC, Mulrow CD, et al. The PRISMA 2020 statement: an updated guideline for reporting systematic reviews. BMJ 2021;372:n71. doi: 10.1136/bmj.n71

For more information, visit: http://www.prisma-statement.org/
